# Comorbidities and Complications in Adult Peritonsillar Abscess Tonsillectomy Patients

**DOI:** 10.1055/s-0045-1810077

**Published:** 2025-10-16

**Authors:** Erin M. Gawel, Alexandra F. Corbin, Gaayathri Varavenkataraman, Sean Clausen, Michele M. Carr

**Affiliations:** 1 Jacobs School Medicine and Biomedical Sciences, University at Buffalo, Buffalo, New York, United States; 2 Department of Otolaryngology, Jacobs School Medicine and Biomedical Sciences, University at Buffalo, Buffalo, New York, United States

**Keywords:** peritonsillar abscess, recurrent tonsillitis, chronic tonsillitis, quinsy tonsillectomy, postoperative complications

## Abstract

**Introduction:**

Tonsillectomy is often used to treat recurrent tonsillitis (RT), but it is less commonly performed to treat peritonsillar abscess (PTA). While most PTAs are treated with needle aspiration or incision and drainage, quinsy tonsillectomy is used in select cases.

**Objective:**

To compare clinical characteristics and postoperative outcomes of patients undergoing quinsy tonsillectomy for PTA versus those undergoing tonsillectomy for RT.

**Methods:**

The American College of Surgeons NSQIP database was used to identify adults who underwent tonsillectomy (CPT code 42826) with a diagnosis of either PTA or RT. Data was collected from 2018–2021. Demographics, comorbidities, risk factors, postoperative complications, and outcomes including operative time, length of stay (LOS), readmission, and reoperation were compared. Logistic regression identified predictors of readmission and reoperation.

**Results:**

10241 patients had RT and 366 had PTA. PTA patients had significantly higher rates of smoking (27.0% versus 12.3%), diabetes (6.6% versus 2.5%), hypertension (11.5% versus 5.8%), and preoperative sepsis (14.5% versus 0.3%; *p* < .001 for all). Operative time and LOS were longer in the PTA group (33.5 minutes versus 25.8 minutes; 2.5 days versus 0.2 days, respectively; *p* < .001 for both). Despite higher rates of rare complications like ventilator use (0.8% versus 0.0%) and sepsis (2.2% versus 0.0%; *p* < .001 for both), no significant differences were observed in postoperative hemorrhage, readmission, or reoperation.

**Conclusion:**

Adults undergoing quinsy tonsillectomy for PTA have more comorbidities and rare complications compared with RT patients, likely due to active infection. However, the procedure is not linked to increased hemorrhage risk and remains safe treatment.

## Introduction

Tonsillectomy is one of the most common surgical procedures performed in otolaryngology. 1 In adults, it is commonly indicated for upper airway obstruction, chronic infection, and peritonsillar abscess (PTA). 2 3 PTA is a collection of pus between the palatine tonsil and the pharyngeal muscles and is the most common deep infection of the head and neck region. 4 5 It is usually caused by bacterial organisms such as Group A Streptococci or Staphylococci, and most frequently occurs in young adults between the ages of 20 and 40 years old. 5 6 Diagnosis of PTA is typically clinical, though computed tomography (CT) and ultrasonography may be used to confirm diagnosis in difficult cases. 5 7 Clinical presentation may include pharyngitis, fever, odynophagia/dysphagia, ipsilateral otalgia, and dysphonia (“hot potato voice”). Uvular deviation and soft palate fullness are also common signs. 7

Standard treatment for PTA includes needle aspiration or incision and drainage under local anesthesia, along with antibiotic therapy and supportive care. 8 9 These procedures are suitable for most PTA patients, with over 90% responding to treatment. However, tonsillectomy may be considered for individuals with a history of persistent PTA or those intolerant of an awake procedure. 9 Quinsy tonsillectomy can be performed at the time of PTA presentation, or a planned interval tonsillectomy may be performed after initial treatment with incision and drainage. 10 11 Previous studies have shown quinsy tonsillectomy to be more advantageous, but indications for each approach remain unclear, and decisions are made based on each patient's clinical situation. 11 12 13

Literature investigating comorbidities and postoperative complications in adults receiving tonsillectomy is limited. We compare clinical profiles and postoperative outcomes in patients undergoing quinsy tonsillectomy for PTA and tonsillectomy for recurrent tonsillitis (RT). Our primary goal was to determine if comorbidities and complication rates were elevated in adults undergoing tonsillectomy to treat PTA.

## Methods

A retrospective analysis was conducted using the American College of Surgeons National Surgical Quality Improvement Program (ACS-NSQIP) database. This national database uses clinical reviewers at participating hospitals to collect data from medical records. Data is collected for 30 days postoperatively and is validated using internal audits. The study was deemed to be nonhuman research and was therefore exempt from approval by the University at Buffalo Institutional Review Board.

The database was searched for CPT code 42826 (tonsillectomy, primary or secondary; age 12 or over) between January 1, 2018, and December 31, 2021. Patients 18 years and older who underwent tonsillectomy as the primary procedure and had a diagnosis of either PTA (ICD-10 code J36) or recurrent, chronic, or acute tonsillitis/pharyngitis (ICD-10 codes J02, J03, J31, J35) were included.

The demographics analyzed included age, sex, and race. Preoperative comorbidities, 30-day postoperative complications, and outcomes of interest were also analyzed. Preoperative comorbidities and risk factors included BMI, diabetes mellitus, smoking, chronic obstructive pulmonary disease (COPD), hypertension, bleeding disorders, systemic sepsis or systemic inflammatory response syndrome (SIRS), and steroid/immunosuppressant use. However, the indication for chronic steroid/immunosuppressant use is not reported by NSQIP. Bleeding disorders were defined according to ACS-NSQIP criteria as any condition associated with an increased risk of excessive bleeding due to impaired clotting mechanisms. This includes patients with documented hematologic disorders such as vitamin K deficiency, hemophilia, thrombocytopenia, von Willebrand disease, and active heparin-induced thrombocytopenia. Additionally, patients on chronic anticoagulation or antiplatelet therapy (excluding aspirin) not discontinued before surgery were classified as having a bleeding disorder. 14 Renal failure and preoperative transfusion were excluded due to small sample sizes. Postoperative complications studied were postoperative hemorrhage, pneumonia, pulmonary embolism, ventilator use, urinary tract infection (UTI), cardiac arrest, bleeding requiring transfusion, and sepsis. Outcomes of interest included operative time, length of hospital stay (LOS), related reoperation, and hospital readmission. Reasons for readmission and reoperation were investigated and categorized based on ICD-10 codes. Three patients remained in the hospital for >30 days post-procedure and were excluded from analyses.

Data analyses were performed using R 4.3.1 (R Core Team, 2023) and SPSS 29.0 (IBM Corp, 2022). Not-reported responses were treated as missing data. Continuous variables were summarized with means, medians, and 95% confidence intervals (CI) and were analyzed using Welch's *t* -tests. Categorical variables were summarized with frequency counts and percentages and were analyzed using Chi-squared tests or Fisher's exact tests if the frequency in any group was less than 5. Logistic regression analysis was performed to estimate the likelihood of readmission and reoperation based on predictor variables. Statistical significance was defined as a p-value <.05.

## Results

### Demographics

A total of 10607 adult patients undergoing tonsillectomy for RT or PTA between January 1, 2018, and December 31, 2021, were included. 10241 patients were in the RT group and 366 were in the PTA group. Demographic information for each patient population can be found in Table 1 . The PTA group had a higher percentage of males ( *N* = 203, 55.5%) than the RT group ( *N* = 3266, 31.9%, *p* < .001). PTA patients were also slightly older than RT patients (31.7 years versus 28.1 years, *p* < .001). There were no racial differences between groups.

**Table 1:** Demographics of patient populations

Demographic	Recurrent tonsillitis/pharyngitis ( *N* = 10241)	Peritonsillar abscess ( *N* = 366)	Total ( *N* = 10607)	*P* -value
Sex, N (%)				<.001 a
Male	3266 (31.9)	203 (55.5)	3469 (32.7)	
Female	6975 (68.1)	163 (44.5)	7138 (67.3)	
Race, N (%)				0.167 b
White	6140 (60.0)	213 (58.2)	6353 (59.9)	
Black or African American	1095 (10.7)	58 (16.0)	1153 (10.9)	
Asian	337 (3.3)	10 (2.7)	347 (3.3)	
Native Hawaiian or Pacific Islander	55 (0.5)	2 (0.5)	57 (0.5)	
American Indian or Alaska Native	39 (0.4)	1 (0.3)	40 (0.4)	
Multiracial	34 (0.3)	0 (0.0)	34 (0.3)	
Unknown or not reported	2541 (24.8)	82 (22.4)	2623 (24.7)	
Mean age in years (95% CI)	28.0 (27.8–28.2)	31.7 (30.3–33.1)	28.1 (27.9–28.3)	<.001 c

a Chi-squared test.

b Fisher's exact test.

c Welsh *t* -test.

### Medical Comorbidities

Medical comorbidities in each patient population can be found in Table 2 . The most common preoperative risk factor was smoking, with nearly 13% of all patients identifying as current smokers. Rates of smoking were significantly higher in the PTA group ( *N* = 99, 27.0% versus *N* = 1259, 12.3%, *p* < .001). The PTA group also had significantly higher rates of diabetes mellitus ( *N* = 24, 6.6% versus *N* = 257, 2.5%, *p* < .001), hypertension ( *N* = 42, 11.5% versus *N* = 589, 5.8%, *p* < .001), and sepsis or SIRS prior to operation ( *N* = 53, 14.5% versus *N* = 29, 0.3%, *p* < .001). Additionally, patients undergoing tonsillectomy for treatment of a PTA also had higher rates of diagnosed bleeding disorders ( *N* = 4, 1.1% versus *N* = 16, 0.2%, p = .004). Other comorbidities and risk factors found in each study population included COPD and steroid/immunosuppressant use, but these variables were not significantly different.

**Table 2:** Medical comorbidities

Comorbidity (N, %)	Reason for tonsillectomy			
	Recurrent tonsillitis/pharyngitis ( *N* = 10241)	Peritonsillar abscess ( *N* = 366)	Total ( *N* = 10607)	*P* -value
BMI (mean, 95% CI)	29.1 (28.9–29.2)	28.8 (28.1–29.5)	29.1 (28.9–29.2)	.368 a
Diabetes mellitus	257 (2.5)	24 (6.6)	281 (2.6)	<.001 c
Current smoker	1259 (12.3)	99 (27.0)	1358 (12.8)	<.001 c
COPD	20 (0.2)	2 (0.5)	22 (0.2)	.175 b
Hypertension	589 (5.8)	42 (11.5)	631 (5.9)	<.001 c
Steroid or immunosuppressant use for a chronic condition	119 (1.2)	7 (1.9)	126 (1.2)	.210 c
Bleeding disorder	16 (0.2)	4 (1.1)	20 (0.2)	.004 b
Systemic sepsis or SIRS	29 (0.3)	53 (14.5)	82 (0.8)	<.001 c

Abbreviations: COPD, chronic obstructive pulmonary disease; SIRS, systemic inflammatory response syndrome.

a Welsh *t* -test.

b Fisher's exact test.

c Chi-squared test.

### Perioperative Outcomes

Perioperative outcomes are summarized in Table 3 . Operative time and LOS varied significantly across groups. Patients in the PTA group had a longer mean operative time than RT patients (33.5 minutes versus 25.8 minutes, *p* < .001), and LOS was the longest in the PTA group (2.5 days versus 0.2 days, *p* < .0001).

**Table 3:** Perioperative outcomes

Perioperative outcomes	Reason for tonsillectomy			
	Recurrent tonsillitis/pharyngitis ( *N* = 10241)	Peritonsillar abscess ( *N* = 366)	Total ( *N* = 10607)	*P* -value
Operative time (min) c	26 (25–26)	34 (32–35)	26 (26–26)	<.001 b
Length of hospital stay (days) c	0.2 (0.1–0.2)	1.0 (0.7–1.2)	0.2 (0.2–0.2)	<.001 b
Related readmission (N, %)	253 (2.5)	7 (1.9)	260 (2.5)	.498 a
Related reoperation (N, %)	371 (3.6)	18 (4.9)	389 (3.7)	.195 a

a Chi-squared test.

b Welsh *t* -test.

c Mean and 95% CI.

There were no significant differences in readmission or reoperation rates between groups. PTA was not predictive of readmission (OR = 1.41, 95% CI: 0.62–3.19, p = .411) or reoperation (OR = 1.05, 95% CI: 0.59–1.84, p = .877). Overall, 325 patients experienced hospital readmissions related to the primary procedure, while 389 required reoperations, most commonly occurring on postoperative days 6.5 and 6.3, respectively. Postoperative hemorrhage was the most common reason for readmission ( *N* = 225, 69.2%) and return to the operating room ( *N* = 354, 91.0%), typically presenting on postoperative day 5.9 (95% CI: 5.1–6.7). A complete list of reasons for readmission and reoperation can be found in Table 4 . Of note, the number of reoperations exceeds the number of readmissions, likely reflecting cases in which patients required surgical intervention before discharge and therefore did not meet the criteria for readmission.

**Table 4:** Reasons for readmission and reoperation

Readmission reason ( *N* = 260)	N, %
Postprocedural hemorrhage	147 (56.5)
Acute postprocedural pain	26 (10.0)
Wound disruption	15 (5.8)
Pneumonia	9 (3.5)
Dehydration	7 (2.7)
Localized edema	5 (1.9)
Surgical site infection	5 (1.9)
Viral infection	3 (1.2)
Dysphagia	3 (1.2)
Acute pharyngitis	3 (1.2)
Nausea and vomiting	2 (0.8)
Pneumothorax	1 (0.4)
Sepsis	1 (0.4)
Pulmonary embolism	1 (0.4)
Unspecified	32 (12.3)
**Reoperation reason (** ***N*** ** = 389)**	**N, %**
Postprocedural hemorrhage	354 (91.0)
Peritonsillar abscess	1 (0.3)
Unspecified	34 (8.7)

Readmission and reoperation did not differ significantly between groups.

Logistic regression analysis showed that demographic variables, comorbidities, and postoperative complications were not associated with readmission. Reoperation was associated with male sex (OR = 2.40, 95% CI: 1.94–2.97, *p* < .001), hypertension (OR = 1.78, 95% CI: 1.15–2.75, p = .009), and bleeding disorders (OR = 9.65, 95% CI: 3.08–30.24, *p* < .001; Table 5 ). Asian patients were significantly less likely to undergo reoperation compared with White patients (OR = 0.60, 95% CI: 0.40–0.89, p = .010; Table 5 ). No differences were found between other races.

**Table 5:** Logistic regression analysis of reoperation

			95% CI		
Variable	Category	Adjusted Odds Ratio	Lower	Upper	*P* -value
Sex	Female	Ref.			
	Male	2.40	1.94	2.97	< 0.001
Race	White	Ref.			
	Asian	0.60	0.40	0.89	0.010
Comorbidities					
Hypertension		1.78	1.15	2.75	0.009
Bleeding disorder		9.65	3.08	30.25	< 0.001

Significant results of binary logistic regression are displayed. Variables included were sex, race, age, BMI, reason for tonsillectomy, diabetes mellitus, current smoker, COPD, hypertension, steroid/immunosuppressant use for a chronic condition, bleeding disorder, systemic sepsis or SIRS, total operation time, and length of hospital stay.

### Postoperative Complications

Postoperative complications are listed in Table 6 . Despite rare complications, ventilator use, and sepsis occurred at higher rates in the PTA group than the RT group ( *p* < .001 for both). 1 patient in the PTA group entered cardiac arrest 15 days after the primary procedure (p = .035). Rates of postoperative hemorrhage, pneumonia, pulmonary embolism, UTI, and bleeding requiring transfusion did not differ between groups.

**Table 6:** Postoperative complications

Postoperative complications (N, %)	Reason for tonsillectomy			
	Recurrent tonsillitis/pharyngitis ( *N* = 10241)	Peritonsillar abscess ( *N* = 366)	Total ( *N* = 10607)	*P* -value
Postprocedural hemorrhage	332 (3.2)	14 (3.8)	346 (3.3)	0.537 a
Pneumonia	20 (0.2)	2 (0.6)	22 (0.2)	0.175 b
Pulmonary embolism	1 (0.0)	0 (0.0)	1 (0.0)	1 b
On ventilator	2 (0.0)	3 (0.8)	5 (0.0)	<0.001 b
Urinary tract infection	36 (0.4)	0 (0.0)	36 (0.3)	0.636 b
Cardiac arrest	0 (0.0)	1 (0.3)	1 (0.0)	0.035 b
Bleeding requiring transfusion	6 (0.1)	1 (0.3)	7 (0.1)	0.218 b
Sepsis	3 (0.0)	8 (2.2)	11 (0.1)	<0.001 b

a Chi-squared test.

b Fisher's exact test.

## Discussion

PTA is associated with various factors that may contribute to its development. First, there is a notable sex disparity, with PTA being more common in males than females. In a retrospective chart review, Bottin et al reported that out of 83 PTA cases, 55 (66.3%) occurred in male patients. 15 Similarly, Slouka et al found that within a cohort of 614 patients aged 19–50 years old, 61.6% were male ( *p* < .0001). 16 The present study findings align with the current literature, as there were more males than females in our PTA study group.

Second, the relationship between diabetes mellitus and PTA has been explored in previous studies. While Wu et al demonstrated a significant correlation between type 2 diabetes mellitus and PTA, similar to the findings in our study. 17 Our data also supports a connection between smoking history and the risk of PTA. Studies by Klug et al revealed that daily tobacco smoking elevates the risk of PTA development by ∼150%, emphasizing the role of smoking in PTA incidence. 18 Similarly, in a cohort study involving over 1,000 PTA patients, Kim et al found higher rates of smoking and alcohol consumption in the PTA group compared with the control group. 19

Hypertension emerges as another association with PTA, supported by our data and studies in the literature. Almutairi et al identified hypertension as a disorder commonly associated with deep neck space infections. 20 Furthermore, several other disorders have been identified as potential correlates with PTA incidence, including renal disease and rheumatoid arthritis. 21 22 23 24 These diverse risk factors highlight the multifaceted nature of PTA development and underscore the importance of considering various health conditions in understanding and managing PTA.

As with any surgical procedure, tonsillectomy in adult patients carries a risk of postoperative complications. Seshamani et al conducted a comprehensive study involving over 35,000 adult patients (ages 18–65) who underwent tonsillectomy for various indications in an outpatient setting. Their findings revealed that 19.7% of patients experienced at least one complication within 14 days of the procedure. Complications included general pain (11.5%), postoperative hemorrhage (6.2%), and dehydration (2.0%). In their study, adult patients with comorbidities, prior history of PTA, or antibiotic overuse were significantly more likely to develop these complications. 25

In our study, adults undergoing tonsillectomy for PTA had more complications than those opting to undergo tonsillectomy for RT. The fact that they are acutely ill likely relates to the complications we found. Although rare, ventilator use, and sepsis were significantly higher in the PTA group post-procedure. These results are expected, considering tonsillectomy is not a first-line treatment for PTA, and individuals may undergo the procedure after unsuccessful needle aspiration or incision and drainage. 2 5 26

We found no increased risk of postoperative hemorrhage in the PTA group compared with the RT group. These findings align with Lehnerdt et al's investigation of 661 tonsillectomy patients, both adults and children, which showed no increased risk of postoperative hemorrhage in abscess tonsillectomies compared with elective tonsillectomies. 27 Further, a study involving 1100 tonsillectomy patients with PTA found no significant difference between the incidence of postoperative bleeding in PTA and RT groups. 28 This underscores quinsy tonsillectomy as a potentially feasible treatment option for adult patients with PTA.

Lastly, the presence of PTA may increase operative time and LOS. This data may be explained by the more complex nature of PTA cases, arising from the presence of active infections and higher rates of preoperative comorbidities among patients. These factors may have broad implications for operating room flow and hospital planning.

### Limitations

Although the ACS-NSQIP database enables analysis of large patient populations, it has several inherent limitations. Notably, it lacks standardized reporting of postoperative pain scores, such as Visual Analog Scale measurements, and does not provide detailed information on analgesic use. This restricts our ability to assess and compare postoperative symptom burden across groups, a key consideration, as pain management can significantly influence outcomes such as readmission and LOS. Additionally, 6% of readmissions and 9% of reoperations are listed for unspecified reasons, reflecting incomplete data fields that are characteristic of large, multicenter databases. Other limitations include the retrospective nature of the study, the potential for data entry errors, and the restricted number of variables collected by NSQIP. Furthermore, because the database includes only NSQIP-participating hospitals and predominantly large academic centers, there may be limited generalizability to other practice settings. Lastly, outcomes beyond 30 days postoperatively are not captured, which may omit important longer-term complications or trends.

## Conclusion

While adults undergoing quinsy tonsillectomy for PTA often present with a greater comorbidity burden and experience higher rates of rare postoperative complications, the procedure is not associated with increased postoperative hemorrhage. Quinsy tonsillectomy remains a safe and effective treatment option, particularly for patients who can tolerate a longer procedure and hospitalization. These findings emphasize the importance of thorough perioperative assessment and planning in this higher-risk population.
